# Eicosapentaenoic acid/docosahexaenoic acid 1:1 ratio improves histological alterations in obese rats with metabolic syndrome

**DOI:** 10.1186/1476-511X-13-31

**Published:** 2014-02-11

**Authors:** Núria Taltavull, Mònica Muñoz-Cortés, Laura Lluís, Montserrat Jové, Àngels Fortuño, Eunice Molinar-Toribio, Josep Lluís Torres, Manuel Pazos, Isabel Medina, M Rosa Nogués

**Affiliations:** 1 Unit of Pharmacology, Faculty of Medicine and Health Sciences, Rovira i Virgili University, Reus, Spain; 2 Unit of Human Anatomy, Faculty of Medicine and Health Sciences, Rovira i Virgili University, Reus, Spain; 3 Anatomical Pathology Service, Eldine Laboratory, Tarragona, Spain; 4 Institute of Advanced Chemistry of Catalonia (IQAC-CSIC), Barcelona, Spain; 5 Institute of Marine Research-CSIC, Vigo, Spain

**Keywords:** Metabolic syndrome, Omega-3-polyunsaturated fatty acids, EPA/DHA ratio, Histology, SHROB rats, Fish oils

## Abstract

**Background:**

Marine polyunsaturated fatty acids, eicosapentaenoic acid (EPA) and docosahexaenoic acid (DHA) have been associated with improvement in the Metabolic Syndrome (MS). The aim of this study is to evaluate how three fish-oil diets with different eicosapentaenoic acid/docosahexaenoic acid ratios (EPA/DHA ratio) affect the histology of liver, kidney, adipose tissue and aorta in a preliminary morphological study. This work uses an animal model of metabolic syndrome in comparison with healthy animals in order to provide information about the best EPA:DHA ratio to prevent or to improve metabolic syndrome symptoms.

**Methods:**

35 Wistar rats, as a control, and 35 spontaneously hypertensive obese rats (SHROB) were fed for 13 weeks with 3 different suplemmentation of fish oil containing EPA and DHA ratios (1:1, 2:1 and 1:2, respectively). All samples were stained with haematoxylin/eosin stain, except aorta samples, which were stained also with Verhoeff and van Gieson’s stain. A histological study was carried out to evaluate changes. These changes were statistically analyzed using SPSS IBM 19 software. The quantitative data were expressed by mean ± SD and were compared among groups and treatments using ANOVA with post-hoc tests for parametric data and the U-Mann–Whitney for non-parametric data. Qualitative data were expressed in frequencies, and compared with contingency tables using χ^2^ statistics.

**Results:**

EPA:DHA 1:1 treatment tended to improve the density and the wrinkling of elastic layers in SHROB rats. Only Wistar rats fed with EPA:DHA 1:1 treatment did not show mast cells in adipose tissue and has less kidney atrophy. In both strains EPA:DHA 1:1 treatment improved inflammation related parameters in liver and kidney.

**Conclusions:**

EPA:DHA 1:1 treatment was the most beneficial treatment since improved many histological parameters in both groups of rats.

## Background

Fish oils are the most common source of Omega-3 fatty acids, mainly eicosapentaenoic acid, (EPA 20:5) and docosahexaenoic acid, (DHA 22:6). It has been pointed out protective effect and beneficial effects of these fatty acids on heart health, cardiovascular disease (CVD), blood lipid profile, Type 2 Diabetes mellitus (T2DM), inflammatory and renal diseases and other illnesses [1,2]. Other potential mechanisms of cardiovascular protection by EPA/DHA may include lowering blood pressure, reduced anti-inflammatory effects, improved insulin resistance and decrease oxidative stress [3,4].

Most of these signs and illnesses are related to metabolic syndrome (MS). MS is defined as the presence of at least three of the five cardiovascular disease risk factors in the following list: elevated fasting triglycerides (TG), decreased high-density lipoprotein cholesterol, insulin resistance, abdominal obesity and high blood pressure [3,5]. Accordingly nutritional modifications such as fish oil supplementation may correct MS.

Physiological changes should lead to morphological and histological changes too, but little research has been done on this issue [6].

Additionally, the amount of n-3 PUFA that needs to be in the diet to provide healthy effects is still unknown [7]. All fish contain EPA and DHA in amounts that vary depending on species. The EPA/DHA ratio in fish ranges between 0.22 and 1.25. The majority of the clinical studies carried-out to date use fish-oil derived dietary supplements, but with a higher EPA/DHA ratio than that commonly found in the fish themselves. There is some controversy about the amounts of DHA or EPA that can have a positive effect on the prevention of metabolic alterations and cardiovascular disease (CVD) [8].

Therefore, the aim of this study is to evaluate how three fish-oil treatments with different EPA/DHA ratios affect the histology of liver, kidney, adipose tissue and aorta in a preliminary morphological study. This paper uses an animal model of metabolic syndrome in comparison to healthy animals in order to provide information about the best EPA: DHA ratio to prevent or to improve MS symptoms.

## Results

### Aorta

**Figure 1:**
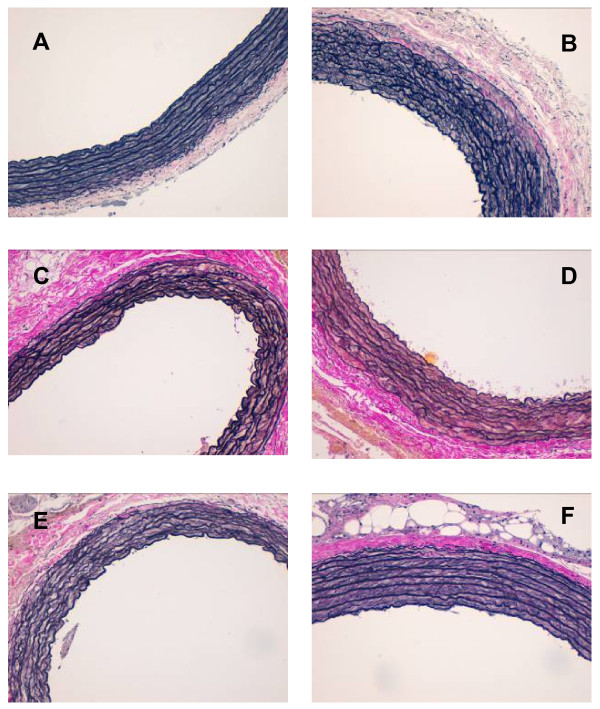
**Aortic wall thickness and wrinkling of elastic layers.** Aortic wall thickness and wrinkling of elastic layers. Venhoeff & Van Gienson stain. (400x). Images on the left: Wistar rats. Images on the right: SHROB rats. **(A and B)** EPA:DHA 1:1 treatment. **(C and D)** EPA:DHA 2:1 treatment. **(E and F)** EPA:DHA 1:2 treatment. As we can see, images on the right, are thicker and less dense, and have less wrinkling, therefore are less functional.

**Figure 2:**
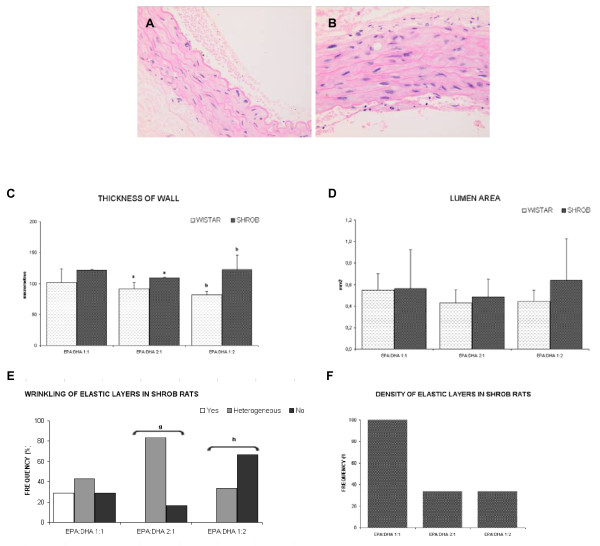
**Aorta results.** At the top Histological cuts of abdominal aorta, Hematoxilin-eosin stain (400x) **(A and B)** EPA:DHA 1:1 in Wistar rat and SHROB rat respectively. We can observe the wall of aorta with the muscular cells nucleus and intuit elastic bands. As we can see SHROB rats presents a nuclei hypertrophy of aortic muscle cells than Wistar rats aortic muscle cells. When there was a nuclear hypertrophy of aortic muscle cells, the size of the cells increased and, consequently, the density decreased and the elastic layers were less wrinkled. This indicates a decreased health status. At the bottom four graphics **(C)** The different treatments did not affect the thickness of the aortic wall in either Wistar or SHROB rats. The same letters in different strains mean significant statistical differences. p = 0.023^a^, p < 0.001^b^. **(D)** In aortic lumen area there were no differences between Wistar and SHROB for any treatment or among treatments in each strain. This indicates that the portion of aorta was taken from the same zone, which enables results to be compared. **(E)** Wrinkling was significantly higher in Wistar than SHROB for the EPA:DHA 2:1, EPA:DHA 1:2 treatments (p = 0.002^g^ and p = 0.002^h^ respectively). The same letters mean significant statistical differences in different strains. **(F)** SHROB rats showed less density than Wistar without significant differences. In SHROB rats group the best value was given by EPA:DHA 1:1 treatment.

Results in the aorta are shown in Table 1 and Figures 1 and 2.

**Table 1:** Aorta results

		**Thickness of wall (μm)**	**Lumen area (mm2)**	**Density of elastic tissue**	**Wrinkling of elastic layers**
		**mean ± SD**	**mean ± SD**	**Yes (%)**	**Yes (%)**
WISTAR	EPA:DHA 1:1	101.60 ± 22.5	0.55 ± 0.31	42.86	85.71
	EPA:DHA 2:1	91.91 ± 9.91^a^	0.43 ± 0.13	57.14	100^c^
	EPA:DHA 1:2	81.66 ± 5.47^b^	0.45 ± 0.10	100*	100^d^
SHROB	EPA:DHA 1:1	121.71 ± 18.04	0.56 ± 0.19	100	28.57
	EPA:DHA 2:1	109.39 ± 13.82^a^	0.49 ± 0.13	33.33	0.00^c^
	EPA:DHA 1:2	122.82 ± 16.68^b^	0.64 ± 0.39	33.33	0.00^d^

The quantitative data are expressed by mean ± SD. Qualitative data are expressed as frequencies.

(*) Statistical differences with respect to EPA:DHA 1:1 treatment p < 0.05.

The same letters in different strains mean significant statistical differences. p = 0.023^a^, p < 0.001^b^, p = 0.002^c^, p = 0.002^d^.

The aortic walls in SHROB rats are less healthy than those in Wistar rats: they are thicker and less dense, and have less wrinkling. However, there is some improvement in those rats consuming a EPA: DHA 1:1 treatment. Both the density and wrinkling are greater in the SHROB group supplemented with this proportion than with the other treatments. Likewise, the thickness of the wall is no different in this SHROB group than in the same Wistar group.

No relationship was found between the anatomopathological findings and oxidative stress. However, aortic wall thickness was positively correlated with total plasma cholesterol levels, and negatively correlated with the HDL/LDL ratio (R^2^ = 0.613 and R^2^ = 0.658, respectively).

### Adipose tissue

**Figure 3:**
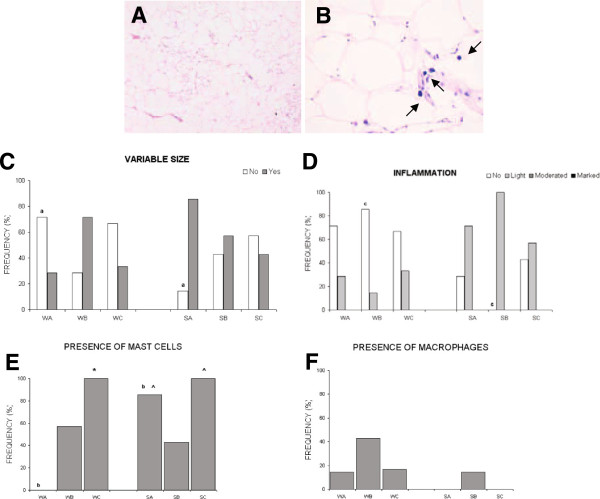
**Adipose tissue results.** At the top Histological cuts of adipose tissue, Hematoxilin-eosin stain **(A)** EPA:DHA 1:1 in SHROB rat (100x), we can observe the variable size of adipocytes and intuit the presence of inflammatory cells. **(B)** EPA:DHA 1:2 in SHROB rat (300x) mast cells marked up by black arrows. At the bottom four graphics in which: (W) refers to Wistar strain, (S) refers to SHROB strain. (A) EPA:DHA 1:1, (B) EPA:DHA 2:1, (C) EPA:DHA 1:2. Equal letters, a, b, c, d, e means statistical differences among strains. **(C)** In both strain of rats there weren’t statistically significant differences in the proportion of different adipocyte sizes among the treatment groups. Comparing the two strains, it was higher in SHROB rats than in WISTAR rats for the EPA:DHA 1:1 diet (p = 0.031^a^). **(D)** As far as the presence of white cells, and therefore inflammation, is concerned; there was a higher effect of PUFA n-3 in Wistar rats than in SHROB rats. In these strain only EPA:DHA 1:2 treatment doesn’t show a significant improvement (p = 0.001^c^) **(E)** Mast cells % in Wistar rats showed differences with EPA:DHA 1:1 (p < 0.001^*^). And in SHROB rats (p = 0.022^^^, p = 0.018^^^ respectively) Comparing the two strains, in the EPA:DHA 1:1 diet, SHROB showed a greater presence of mast cells than WISTAR (p = 0.001^b^). **(F)** The proportion of macrophages in SHROB rats was lower than in Wistar rats but no significant differences were founded.

Results in adipose tissue are shown in Table 2 and Figure 3.

**Table 2:** Adipose tissue results

		**Variable size**	**Macrophages**	**Mast cells**	**Inflammation**			
		**Yes (%)**	**Yes (%)**	**Yes (%)**	**No (%)**	**Light (%)**	**Moderate (%)**	**Marked (%)**
WISTAR	EPA:DHA 1:1	28.57^a^	14.29	0^b^	71.43	28.57	0.00	0.00
	EPA:DHA 2:1	71.43	42.86	57.14	85.71^c^	14.29	0.00	0.00
	EPA:DHA 1:2	33	16.67	100*	66.67	33.33	0.00	0.00
SHROB	EPA:DHA 1:1	85.71^a^	0.00	85.71^^b^	28.57	71.43	0.00	0.00
	EPA:DHA 2:1	57.14	14.30	42.86	0.00^c^	100.00	0.00	0.00
	EPA:DHA 1:2	42.86	0.00	100^	42.86	57.14	0.00	0.00

Data are expressed as frequencies. The same letters in different strains mean significant statistical differences, p = 0.031^a^, p = 0.01^b^, p = 0.001^c^.

(*): Differences with EPA:DHA 1:1. In mast cells % p < 0.001. (^): differences with EPA:DHA 2:1. In mast cells % p = 0.022, p = 0.018 respectively.

To sum up, SHROB rats present greater hypertrophy and inflammation of the adipose tissue than Wistar rats. The results for each treatment group show that the 1:1 ratio presented no mast cells in Wistar rats unlike other groups. The 2:1 treatment decreased the number of mast cells in the adipose tissue of SHROB rats with respect to the other treatments. And, the 1:1 and 1:2 treatments showed no macrophages in SHROB rats unlike the other groups.

### Liver

**Figure 4:**
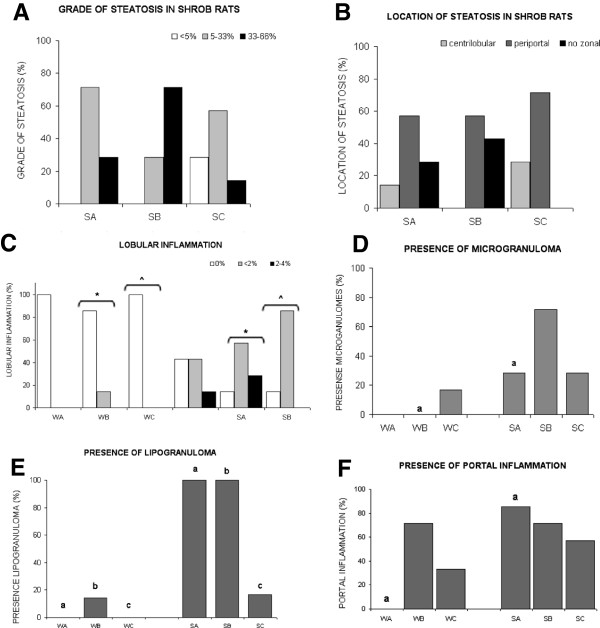
**Liver results.** Graphics of liver results in which: (W) refers to WISTAR stain, (S) refers to SHROB stain. (A) EPA:DHA 1:1, (B) EPA:DHA 2:1,(C) EPA:DHA 1:2. Equal letters, a, b, c, d, e means statistical differences among strains. **(A)** Liver samples of WISTAR rats no showed steatosis. Consequently, between WISTAR and SHROB statistically significant differences were observed in all treatment groups (EPA:DHA 1:1 p = 0.001, EPA:DHA 2:1 p = 0.001, EPA:DHA 1:2 p = 0.031). The graphics showns the grade of stetosi in SHROB rats, its location is shown in graphic **(B)** we can see how in SHROB rats steatosis tended to be periportal, but no statistically significant differences were found between treatment groups. **(C)** SHROB rats also showed greater lobular inflammation than Wistar. There were significant differences between strains in the EPA:DHA 2:1 and EPA:DHA 1:2 treatments (p = 0.025* and p = 0.02^^^, respectively). **(D)** The presence of microgranulomas was higher in SHROB rats than in Wistar, and the differences were significant in three tretament groups: EPA:DHA 2:1 (p = 0.005a). In SHROB fewer microgranulomas were present in the EPA:DHA 1:1 and EPA:DHA 1:2 treatments, but there were no significant differences with other groups. **(E)** We found lipogranulomas in the Wistar EPA:DHA 2:1 group and in all the groups of SHROB rats, with significant differences between strains for EPA:DHA 1:1 (p < 0.001), EPA:DHA 2:1 (p = 0.001), EPA:DHA 1:2 (p = 0.002). **(F)** Portal inflammation was present in both Wistar and SHROB rats but differences were only significant between strains in the EPA: DHA 1:1 treatment (p < 0.001). No differences between treatments were found in the same strain.

**Figure 5:**
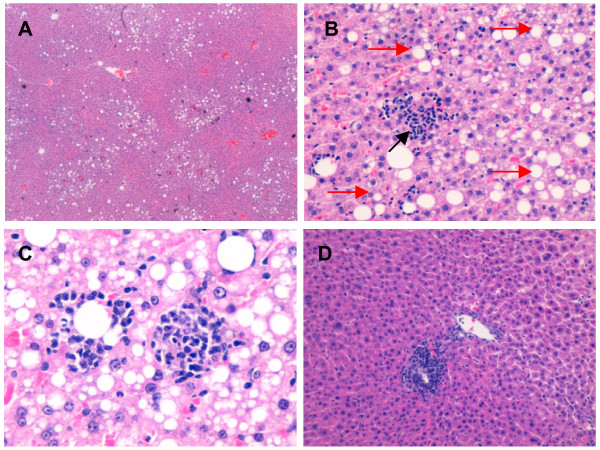
**Histological cuts of liver.** Histological cuts of liver, Hematoxilin-eosin stain **(A)** EPA:DHA 2:1 treatment in SHROB rat (40x), shown steatosis with periportal location **(B)** EPA:DHA 2:1 treatment in SHROB rat (400x), lobular inflammation marked up with a black arrow. The picture also allows us to observe steatosi which is marked up, in part, with red arrows **(C)** EPA:DHA 2:1 in SHROB rat (600x), on the right some macrophages surrounding a lipidic drop, forming a lipogrnaulome and on the left a lipogranulome also formed by macrophages. The picture also allows us to observe steatosi at higher magnification **(D)** EPA:DHA 2:1 in Wistar rat (200x), portal inflammation, as we can see the inflammatory cells are surrounding portal space.

Results are shown in Figures 4 and 5. To sum up, in liver, the presence of steatosis in the centrilobular zone indicates initial harmful effects. The more widespread it is, the more the injury has progressed and the more severe it is. Therefore, periportal steatosis is worse than centrilobular steatosis, and non-zonal inflammation is the worst. In SHROB rats steatosis tended to be periportal, but no statistically significant differences were found between treatment groups. As in the other tissues, inflammation was evaluated according to the presence of white cells. SHROB rats also showed greater lobular inflammation than Wistar.

### Kidney

**Figure 6:**
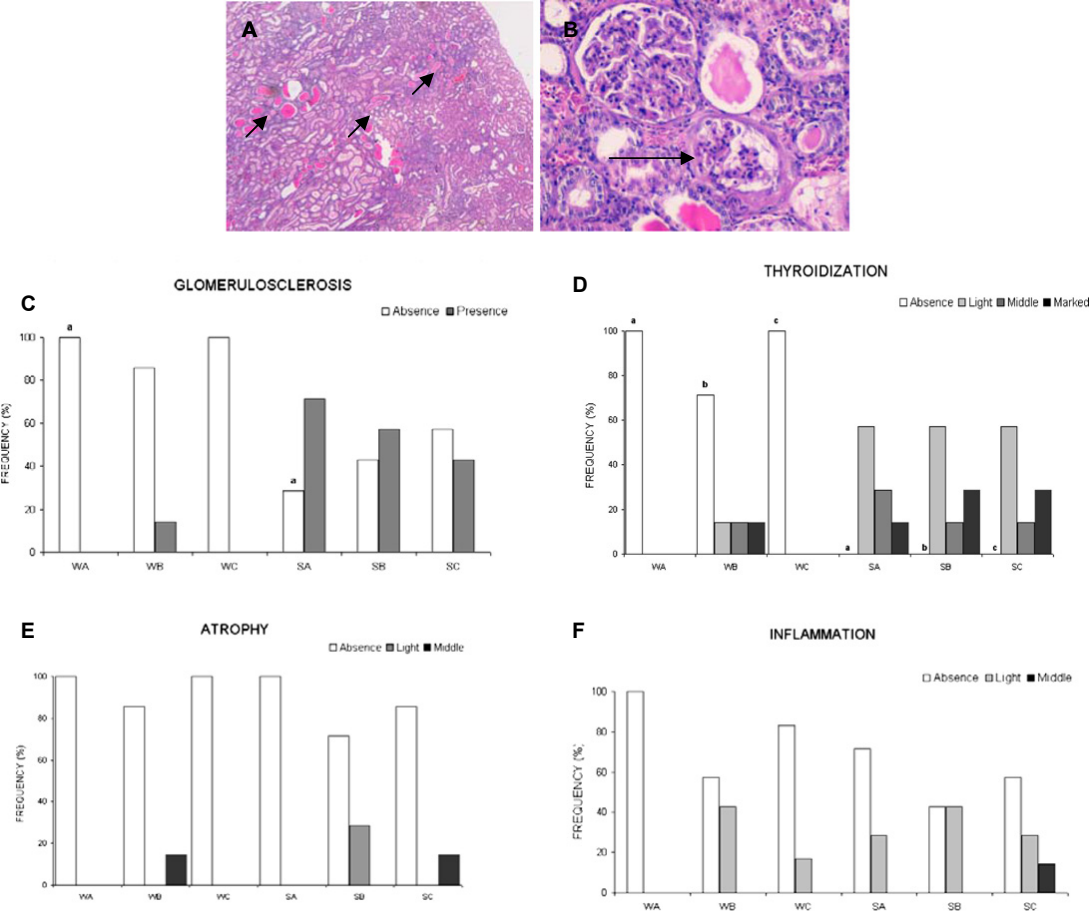
**Kidney results.** At the top Histological cuts of kidney, Hematoxilin-eosin stain. **(A)** EPA:DHA 1:1 in SHROB rat (40x) thyroidization zones are marked by black arrows. Thyroidization it’s a kind of atrophy which gives them the appearance of the follicles in the thyroid gland as we can see in the picture (hence the name). **(B)** EPA:DHA 1:2 in SHROB rat (200x), inflammation in kidney can be observed by the presence of inflammatory cells, also we can observe a normal glomerulus on the top and a glomerulus with glomerulosclerosis marked by a black arrow. At the bottom three graphics in which: (W) refers to Wistar strain, (S) refers to SHROB strain. (A) EPA:DHA 1:1, (B) EPA:DHA 2:1, (C) EPA:DHA 1:2. Equal letters, a, b, c, d, e means statistical differences among strains. **(C)** We found greater presence of glomerulosclerosis in SHROB rats than in Wistar rats, but significant differences were found only in the EPA:DHA 1:1 treatment (p = 0.005^a^). **(D)** SHROB rats had greater thyroidization than Wistar rats in all treatment groups p = 0.003^a^, p = 0.032^b^, p = 0.005^c^. **(E)** Atrophy was also higher in SHROB rats. EPA:1:1 doesn’t shown atrophy in either both treatments, but no significant differences were founded. **(F)** Inflammation were better also in EPA:DHA 1:1 treatments in both strains, but without significant differences.

Table 3 and Figure 6 shows the results of the different variables studied in kidney. To sum up, kidney damage was greater in SHROB than in Wistar rats. The 2:1 treatment tends to worsen the kidney parameters in Wistar rats, while the 1:1 treatment improves renal atrophy in SHROB in comparison to the other tretments. The EPA:DHA 1:1 treatment had the lowest presence of atrophy followed by EPA:DHA 1:2. There were no differences in fibrosis, lipid depositions or inflammation of the kidney either between treatments or between strains.

**Table 3:** Kidney results

		**Glomerulosclerosis**	**Atrophy**				**Thyroidization**			
		**Presence**	**Absence**	**Light**	**Middle**	**Marked**	**Absence**	**Light**	**Middle**	**Marked**
WISTAR	EPA:DHA 1:1	0^a^	100	0	0	0	100^a^	0	0	0
	EPA:DHA 2:1	14.29	85.71	0	14.29	0	71.43^b^	14.29	14.29	14.29
	EPA:DHA 1:2	0	100	0	0	0	100^c^	0	0	0
SHROB	EPA:DHA 1:1	71.43^a^	100	0	0	0	0^a^	57.14	28.57	14.29
	EPA:DHA 2:1	57.14	71.43	28.57	0	0	0^b^	57.14	14.29	28.57
	EPA:DHA 1:2	42.86	85.71	0	14.29	0	0^c^	57.14	14.29	28.57

The data are expressed as frequency. The same letters in different strains mean significant statistical differences.

Glomerulosclerosis: p = 0.005^a^.

Thyroidization: p = 0.003^a^, p = 0.032^b^, p = 0.005^c^.

## Discussion

Although the relation between PUFA and cardiovascular disease has been widely demonstrated [1,9,10], limited information is available for histological changes.

### Aorta

Data on various aortic layers are essential for understanding various diseases (such hypertension or diabetes) that may remodel the tissue geometry and biomechanical properties of the vascular wall differentially [11].

Only two previous studies have investigated the effect of marine fatty acids on aorta histopathology. Park S. and Park Y. results suggested that the hypolipidemic action of fish oil had protective effects on aorta histopathology in male Wistar rats [12]. And Engler, *et al.*, who show how DHA-fed spontaneously hypertensive rats significantly reduced blood pressure and vascular wall thicknesses in the coronary, thoracic, and abdominal aorta compared with controls [13].

In our study, despite the thickness of the aorta was not affected by any of the treatments, the ratio EPA:DHA 1:1 shown the highest value. And the highest value of wrinkling too. This indicates a healthier condition. A lower density of elastic layers and less wrinkling indicates increased stiffness of the aorta which has been associated with greater vascular damage [11,14].

That’s the reason why the ratio EPA:DHA 1:1 is the best at maintaining or improving the aorta’s structure, and hence its function. We should emphasize that the portion of the aorta was taken from the same zone, therefore the results of the other variables can be attributed to diet or strain and not to the location of the tissue sampling. All treatments had a similar fat and energy content and, hence, the observed differences can be attributed to the different EPA:DHA ratios.

### Adipose tissue

According to the literature, omega 3 supplementation prevents the inflammation of the adipose tissue in obese and diabetic mice [9] and diminishes hypertrophy and hyperplasia in that tissue [15]. Our results seem to be contrary to the literature; in adipose tissue we observed hypertrophy of tissue when the abdominal fat weight/body weight ratio in SHROB rats (6.7-7.8%) was higher than in Wistar rats (3.6-4.3%). There were significant differences in all treatment groups. Treatments did not affect this ratio in either Wistar or SHROB rats. This may be because SHROB rats were too severely affected by the diseases or because the weekly recommended dosage was too low.

The greater presence of differently-sized adipocytes could be explained by the relation between PUFA, and peroxisome-proliferator-activated receptor γ (PPARγ), which plays a critical role in the regulation of adipocyte differentiation. PUFA are potential ligands for this nuclear receptor, which may be the reason why rats fed PUFA have more adipocytes of different sizes [16]. The PPAR family can also inhibit NF-кß factor transcription, which is also related to inflammation [5]. This may explain why PUFA prevents inflammation.

However, the effect of different types of PUFA on body adiposity is still controversial. Omega-3 PUFA treatments, as our results show, improve inflammation in this tissue, especially in SHROB rats. Even being a very affected model, after the EPA:DHA treatment SHROB rats showed a slight inflammation or non- existent inflammation in all cases.

### Liver

The effects of omega-3 PUFA on liver are the most controversial, showing contradictory results [17,18]. In our study Wistar rats showed better results in all the parameters determined in liver, specifically EPA:DHA 1:1 treatment, which showed the best results, leading to a total absence of any inflammatory process.

Various studies have shown that both the excess and the total absence of omega 3 PUFA in experimental animals have given rise to hepatic steatosis [19,20]. This could be explained by the nature of the PUFA n-3, which is fat. It is therefore of the utmost importance to establish the correct dosage range for these fatty acids.

In our study, in healthy rats - Wistar rats- , the best treatment is EPA:DHA 1:1 implying a preventative role in the liver. However in the group of rats affected -SHROB rats- EPA:DHA 1:1 ratio also provides the less harmful location of steatosis.

### Kidney

Finally, epidemiological studies suggest that omega-3 PUFA slow down the progression of renal dysfunction in patients [21,22] and experimental models of diabetes [23,24].

In our results, unlike SHROB rats, Wistar rats showed no renal dysfunction. A comparison of the treatments in SHROB rats shows that EPA:DHA 1:1 did not cause renal atrophy.

Considering the greater effect in Wistar rats than in SHROB rats, treatments of marine fish-oil seem to be more preventative than restorative in kidney tissue. As in liver tissue.

All this suggests that within the protective role of PUFA, the most appropriate ratio of its components is EPA:DHA 1:1 ratio as has been already described in the article of Lluis, *et al.*[4] carried on by our group. But also improves histological parameters in Obese rats with Metabolic Syndrome, as demonstrated in this work.

### Limitations

The SHROB strain, also known as the Koletsky rat, is considered to be a good model for MS. It has monogenetic obesity superimposed on a hypertensive genetic background. The obesity mutation is a recessive trait, designated fa^k^, which is a nonsense mutation of the leptin receptor gene. This mutation renders the SHROB incapable of central and peripheral responses to leptin. Animals can be identified as genetically obese at about five weeks of age and they also develop premature vascular disease, particularly of the abdominal arteries, although, microscopically, the lesions that occur in these vessels simulate those of human atherosclerosis [9]. The SHROB rats also develop severe glomerulosclerosis and renal failure [25]. SHROB strain has been used in many pharmacological studies with antihypertensive and antidiabetic drugs but our results indicate that it is probably not such a useful model for distinguishing subtle changes caused by diets.

## Conclusion

PUFA n-3 treatments – especially EPA:DHA 1:1 ratio– improved the density and wrinkling of the elastic layers in the abdominal aorta in SHROB rats and kidney atrophy as well. EPA:DHA 1:1 also improved the inflammation parameters in the liver and the inflammation parameters in adipose tissue in both groups of rats. Therefore we can conclude that, Eicosapentaenoic acid/Docosahexaenoic acid 1:1 ratio improves histological alterations in Obese Rats with Metabolic Syndrome which is a progress in the study of morphological changes and therefore functional changes of PUFA in the organism. It is important to note that is a preliminary morphological study, therefore most studies had to be done in this field.

## Methods and procedures

### Animals and supplementation

All the procedures agreed with the European Union guidelines for the care and management of laboratory animals and the pertinent permission was obtained from the CSIC Subcommittee of Bioethical Issues.

Thirty-five female Wistar rats, as a control group, and thirty-five spontaneously hypertensive obese rats (SHROB), as a model of SM, were purchased from Janvier (Le Genest-St-Isle, France).The rats (13 weeks old) were kept in an isolated room with a constantly regulated temperature (22 ± 2°C), and controlled humidity (50 ± 10%) in a 12 h. artificial light cycle. Both groups of rats spent a prior period of adaptation that lasted two weeks.

Rats of each strain were randomized into trhee groups (7 rats each): EPA:DHA 1:1 group, EPA:DHA 2:1 group and EPA:DHA 1:2 group. All groups were fed a standard pelleted diet from Harlan Ibérica (Barcelona, Spain) and had ad libitum access to water and food.

Oils with different EPA:DHA ratios were obtained by mixing appropriate quantities of the commercial fish oils AFAMPES 121 EPA (A.F.A.M.S.A., Vigo, Spain), EnerZona Omega 3 RX (Milan, Italy) and Oligen liquid DHA 80% (IFIGEN-EQUIP 98, S.L., Barcelona).

The supplemented dose fish oils was in agreement with the European Union’s recommendation on omega-3 PUFA [26]. All supplements had a similar fat and energy content (55–66 mg of PUFA per 100 mg of total fatty acid) as is specified in a previous study recently published by our group [27]. However, they differed significantly in the proportion of individual fatty acids. In fish oil mixtures containing EPA:DHA ratios of 1:1, 2:1 and 1:2, EPA and DHA were the most abundant PUFA, making up approximately 50 mg per 100 mg of fatty acid between them. Because PUFA are extremely susceptible to oxidation and the potential toxic effects of lipid oxidation by-products, the lipid oxidation level was checked throughout the experiment (peroxide values < 5 meq. oxygen per kg of oil).

Each group of rats (n = 7) had a weekly oral dose of 0.8 mL/Kg bodyweight of the oil supplements, for 13 weeks administered by gavage.

Finally all rats were sacrificed at the age of 28 weeks. The rats were anesthetized intraperitoneally with ketamine and xylacine (80 mg/Kg and 10 mg/Kg body weight respectively), and then killed by exsanguination. Tissue samples were collected from the abdominal aorta, abdominal adipose tissue, liver, and kidney.

### Tissue processing

The dissected tissues were divided into two parts: the first one was quickly frozen in liquid nitrogen and stored at -80°C, for various biochemical determinations, and the second one was fixed in 10% formalin at pH 7.4, for histological analysis. All tissues, except adipose tissue, which was extracted from abdominal zone, were dissected entirely.

The samples fixed in formalin were dehydrated in alcohol and embedded in paraffin. Then all tissues were cut in typically 3 μm thick slices, using a steel Knife mounted in a microtome (Microm HN 355 s). Sections from the liver, kidney and adipose tissue were stained with haematoxylin/eosin (Harris Hematoxylin. QCA). Aorta sections were stained with hematoxylin/eosin, and Verhoeff and van Gieson’s stain (Van Gieson kit 100 test 6 × 30 ml + 2 × 18 ml Casa Alvarez (WVG-1026). Haematoxylin stains cell nuclei blue, while eosin stains cytoplasm, connective tissue and other extracellular substances pink or red. Verhoeff and van Gieson's stain is a mixture of picric and fuchsin acids which makes it possible to observe the elastic layers of the aorta wall by staining them dark blue. The sections of all tissues were viewed under a light digital optical microscope (Leica DMD 108) for histopathological changes.

Results of biochemical determinations such, as tissue oxidative stress and plasma lipid profile are not shown in this study, and were only used to establish correlations with the parameters of pathological anatomy.

The tissue parameters were graded on the basis of the histological findings (Table 4) [12,13,28-30].

**Table 4:** Categorization of the parameters in the different tissues

**Meaning**	**Grade**	**Meaning**	**Grade**	**Meaning**	**Grade**
**Aorta**		**Liver**		**Kidney**	
Thickness of wall (μm)		Steatosis		Glomerulosclerosis	
Lumen perimeter (mm)		< 5%	0	Absence	0
Lumen area (mm^2^)		5-33%	1	Presence	1
Density of elastic bands		33-66%	2	Atrophy	
Yes	1	> 66%	3	Absence	0
No	2	Localization of steatosis		Light	1
Wrinkling of elastic layers		Centrilobular	0	Middle	2
Yes	1	Periportal	1	Marked	3
Heterogeneous	2	No zonal	2	Thyroidization	
No	3			Absence	0
Nuclear hypertrophy		Lobular inflammation		Light	1
No	1	No	0	Middle	2
Heterogeneous	2	< 2 focus	1	Marked	3
Yes	3	2-4 focus	2	Fibrosis	
		> 4 focus	3	Absence	0
**Adipose tissue**		Portal inflammation		Presence	1
Differently sized adipocytes		No	0	Lipid depositions	
Yes	1	Yes	1	Absence	0
No	0	Fibrosis		Light	1
Macrophages		No	0	Moderate	2
Presence	1	Perisinusoidal or periportal	1	Inflammation	
Absence	0	Fibrosis bridges	2	Absence	0
Mast cells		Cirrhosis	3	Light	1
Presence	1	Microgranuloma		Moderate	2
Absence	0	Absence	0	Marked	3
Inflammation		Presence	1		
No	0	Lipogranuloma			
Light	1	Absence	0		
Moderate	2	Presence	1		
Marked	3	Macrophages			
		Absence	0		
		Presence	1		

Categorization of the parameters in the different tissues based on the literature: Park S and Park Y, 2009 [12]; Engler, *et al.*, 2003 [13]; Muñoz, *et al.,* 2009 [28]; Xu ZJ, *et al.*,2010 [29]; Dobrian, *et al.*, 2003 [30].

The thickness of the aortic wall was examined at two different points: maximum and minimum thickness. The mean of these two values was used. Semi quantitative parameters were assessed by the consensus of three observers.

Inflammation of adipose tissue, liver and kidney was determined on the basis of the presence of inflammatory cells in tissue [28,30].

Varying adipocyte sizes indicate hypertrophy in adipose tissue. The more adipocytes there are of different sizes, the greater the degree of tissue hypertrophy [28].As well as inflammation, glomerulosclerosis, atrophy, fibrosis, lipid deposits and thyroidization were also evaluated in kidney.

Thyroidization is the atrophy of some areas of the kidney. The presence of thyroidization, then, does not indicate a healthy status.

The results of the categorical variables were expressed as the percentage of animals that presented the particular category in every variable (relative frequency).

### Statistical analysis

Statistical analysis was performed using SPSS IBM 19 software. The quantitative data are expressed by mean ± SD and were compared among groups and treatments using ANOVA with post-hoc tests for parametric data and the U-Mann–Whitney for non-parametric data. Qualitative data are expressed in frequencies, therefore, percent of animals which present or not present the variable. Relative frequencies were compared with contingency tables using χ^2^ statistics. A p-value of <0.05 was considered to be statistically significant. Results were statistically correlated with oxidative stress and lipid profile data.

## Abbreviations

ANOVA: Analysis of variance; Apo: Apolipoprotein; CDV: Cardiovascular disease; DHA: Docosahexaenoic acid; EPA: Eicosapentaenoic acid; HDL: High-density lipoprotein; LDL: Low-density lipoprotein; MS: Metabolic syndrome; NF-қB: Nuclear factor kappa B; PUFA: Polyunsaturated fatty acids; PPAR: Peroxisome proliferator-activated receptor; SHROB: Spontaneously hypertensive obese rats; SHR: Spontaneous hypertensive rats; VLDL: Very-low-density lipoprotein.

## Competing interests

The authors declare that they have competing interests.

## Authors’ contributions

All the authors have contributed substantially to the design and execution of the study as well as the drafting and revision of the manuscript. They have all approved the final version submitted for publication.
